# Trends in the Use of Pit and Fissure Sealants in Adolescents and Their Impact on the Decayed, Missing, and Filled Teeth (DMFT) Index: An Ecological Study in Mexico

**DOI:** 10.7759/cureus.72064

**Published:** 2024-10-21

**Authors:** Amairany M Torres-Sánchez, Salvador E Lucas-Rincón, América P Pontigo-Loyola, Martha Mendoza-Rodríguez, Sandra I Jimenez-Gayosso, Alejandro J Casanova-Rosado, Juan J Villalobos-Rodelo, Rosalina Islas-Zarazúa, Carlo E Medina-Solís, Gerardo Maupomé

**Affiliations:** 1 Joint Program of Dental Specialties With Emphasis on Pediatric Dentistry, Academic Area of Dentistry of Health Sciences Institute, Autonomous University of Hidalgo State, Pachuca, MEX; 2 Academic Area of Dentistry of Health Sciences Institute, Autonomous University of Hidalgo State, Pachuca, MEX; 3 School of Dentistry, Autonomous University of Campeche, Campeche, MEX; 4 School of Dentistry, Autonomous University of Sinaloa, Culiacan, MEX; 5 Advanced Studies and Research Center in Dentistry, "Dr. Keisaburo Miyata" School of Dentistry, Autonomous University of State of Mexico, Toluca, MEX; 6 Epidemiology, Richard M. Fairbanks School of Public Health, Indiana University, Indianapolis, USA

**Keywords:** dental caries, health services, oral health, pit and fissure sealants, schoolchildren

## Abstract

Background: Untreated dental caries in permanent teeth is the most common health disorder. Pit and fissure sealants (PFS) are one of the effective treatment options that help prevent caries.

Objective: The objective of this study is to evaluate the temporal trends in the use of PFS among adolescents (ages 10-14) in Mexico from 2005 to 2014 and to determine their specific impact on the decayed, missing, and filled teeth (DMFT) index at the national level, using aggregated data from epidemiological surveillance.

Methodology: An ecological study was carried out. The data were from the Epidemiological Surveillance System of Oral Pathologies (SIVEPAB, 2005-2014). The dependent variable was the DMFT index. The independent variable was PFS prevalence during the time of the study. In the statistical analysis, the non-parametric test for trends and Spearman's correlation in Stata were used.

Results: The average DMFT was 3.43 in children 10 to 14 years old. During the 10-year study period, in no year were more than 2.5% of 12-year-old adolescents attending publicly funded dental health services in Mexico observed to have one or more teeth with PFS. The average PFS prevalence use in 12-year-old adolescents was 1.57%. No trend was observed in the use of PFS (z=-0.66, p=0.509), but there was a decrease in the DMFT index (z=-2.54, p=0.011). There was no statistically significant correlation (p>0.05) between the percentage of adolescents with at least one tooth with PFS and the average DMFT.

Conclusion: In the present ecological study, PFS prevalence was low. While there was no discernible trend in PFS use, there was a decrease in the DMFT index, seemingly unrelated to PFS use.

## Introduction

Although most oral diseases are preventable and treatable in their early stages through simple actions, they collectively represent a significant burden on the healthcare system in many countries [1,2]. They may also impact the quality of life of the population together with pain, increased school absenteeism, decreased work performance, cosmetic appearance, and even death. Dental caries, periodontal diseases, tooth loss, and oral cancers account for the majority of these conditions [3,4]. These diseases affect almost 3.5 billion people worldwide. Untreated dental caries in permanent teeth is the most common health disorder according to the Global Burden of Disease Study 2019 [5]. It preferentially affects socially disadvantaged groups with limited access to healthcare services, with significant associated socioeconomic inequalities [1,6,7]. Most low- and middle-income countries do not have sufficient preventive or treatment services for oral health conditions, likely because specialized treatment for these conditions is expensive and is not usually included in universal health coverage, when available [8]. The 74th World Health Assembly approved a resolution on oral health in 2021, recommending moving from the traditional curative approach to the adoption of preventive health promotion, encompassing oral health in the family, school, and workplace [9].

Dental caries is a multifactorial disease derived from the interaction between the oral microbiome, the host (e.g., genetics, age, dental morphology, self-cleaning, and oral hygiene), and a diet with a high content of simple carbohydrates. Dysbiosis alters the oral environment with the proliferation of acidogenic bacteria, the acids derived from their bacterial metabolism, and a resulting lower pH decrease. The cumulative effect of such exposure to acids demineralizes dental tissues, progressively leading to their structural weakening, and eventually manifested clinically as a cavity [10,11]. The deep pits and fissures of the occlusal surfaces of posterior teeth are particularly prone to caries: they account for 90% of caries experienced by children and adolescents, compared to smooth surfaces [12]. Pit and fissure sealants (PFS) are one of the treatment choices that, when applied to occlusal pits and fissures, form a micromechanically bonded physical barrier that prevents bacteria and food particles from getting trapped in the fissures, thus preventing the onset of caries and partially blocking the progression of carious lesions [11,13-15].

In Mexico, dental caries is a public health problem for children [16-19]. The use of PFS is an effective measure for the prevention of pit and fissure caries in children, as documented in various studies [20-22]. Epidemiological studies on the subject define PFS use as a child with at least one sealed tooth. While a study in Italy on 2442 children aged six to 12 reported a PFS prevalence of 2.7%, a study in the USA involving 2220 children and adolescents up to 18 years old reported that 45.5% had received PFS [23,24]. Another study using data from the National Health and Nutrition Examination Survey (NHANES 2005-2010) found that PFS prevalence was 31.3% among American children and adolescents aged five to 18 [25]. There is considerable variation in PFS use across countries, such that in Santo Tomé Island, 1855 children aged 11 to 14 had a 2.2% PFS prevalence; a report from China found a 3.7% PFS prevalence in 504 students [26,27]. Those variations in PFS prevalence exist not only across countries but also across socioeconomic situations; this is a suboptimal situation since the application of PFS is an effective and low-cost measure to prevent caries. PFS prevalence can also be used as a marker of the performance of public health systems because it is reasonable to expect that its widespread use would improve the DMFT index. The objective of this study is to evaluate the temporal trends in the use of PFS among adolescents (aged 10-14) in Mexico from 2005 to 2014 and determine their specific impact on the DMFT index at the national level, using aggregated data from epidemiological surveillance.

## Materials and methods

Study design

An ecological study was conducted on adolescents using publicly funded dental health services in Mexico. An ecological study is characterized by studying groups rather than individuals separately, using data recorded in public institution statistics or open registries, such as national surveys. From 2005 to 2014, the Epidemiological Surveillance System of Oral Pathologies (SIVEPAB) provided such data in Mexico [28]. The Mexican Public Health Sector uses sentinel units to gather information from the SIVEPAB. The data collection instrument is the SIVEPAB 1 oral pathology case study form, which is filled out by trained personnel assigned to dental service units.

Sample

The study population is 12-year-old adolescents (for PFS use) and 10- to 14-year-old adolescents (for the DMFT index). This is a census, as the available data from the SIVEPAB includes the 32 states in Mexico. Data from 2005 to 2014 were used.

Variables

The dependent variable was the DMFT index in adolescents aged 10-14 in the selected years. The independent variable was the percentage of adolescents aged 12 with PFS applied from 2005 to 2014.

Statistical analysis

Graphs and tables were used in the analysis. The graphs and trend lines were generated in Excel, using the scatter plot tool and trend line format. The option to display the equation in the graph and the R-squared value in the graph was also selected. The non-parametric test for trends and Spearman's correlation test in Stata were used to test our hypothesis.

Ethical considerations

The Bioethics and Research Committee of the School of Dentistry of Autonomous University of Campeche (CBI-09-2022-6) granted IRB approval for the retrospective analyses of published health services data. Informed consent forms were not required.

## Results

**Figure 1:**
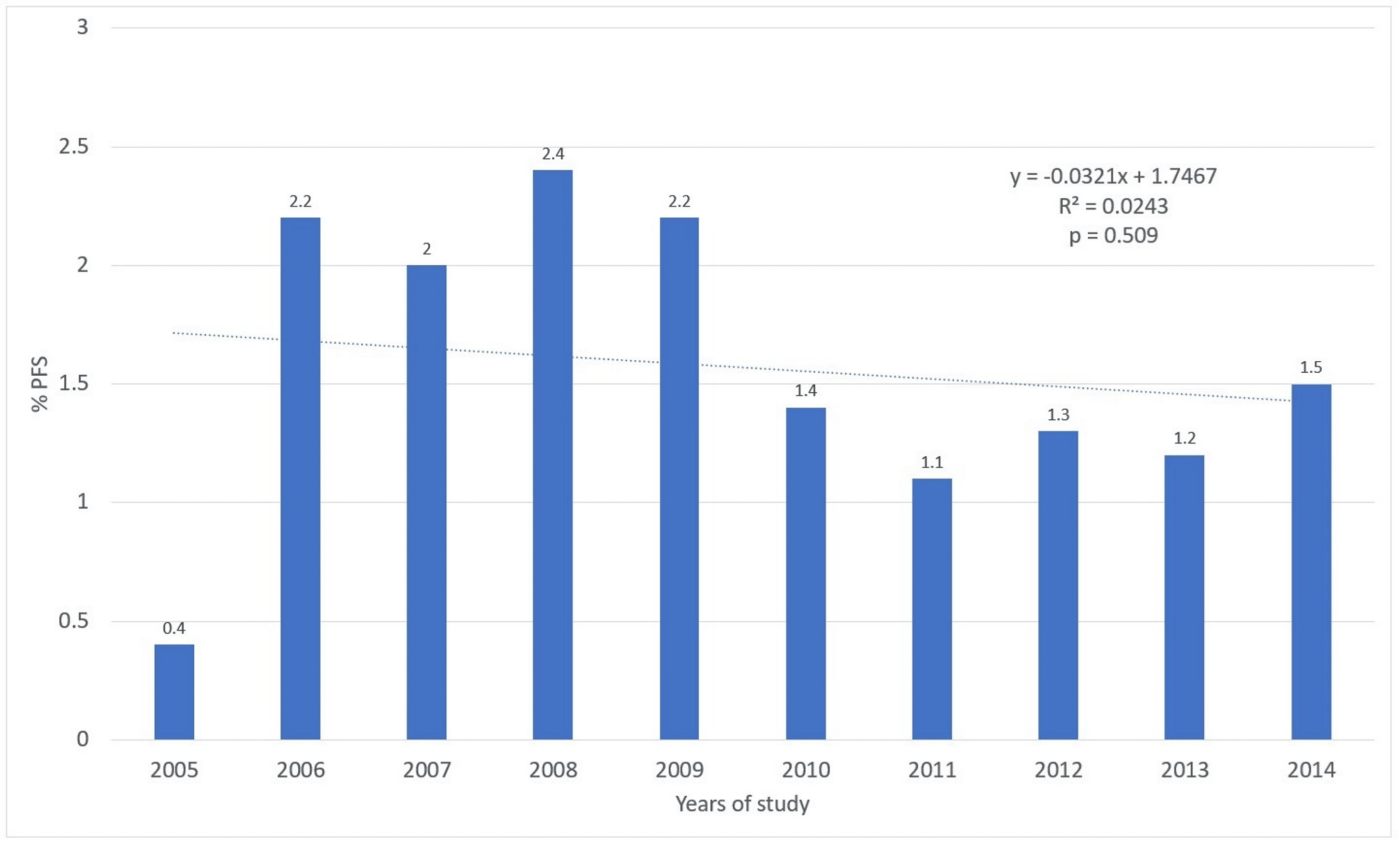
Percentage of 12-year-old adolescents with at least one tooth with PFS per year in Mexico. The p-value was calculated using the nonparametric test for trends across ordered groups. Trend lines were generated in Excel, using the trend line format. PFS, pit and fissure sealants

During the 10-year study period, in no year were more than 2.5% of 12-year-old adolescents attending publicly funded dental health services in Mexico observed to have one or more teeth with PFS. The lowest percentage of adolescents with PFS was in 2005 (0.4%), and the highest percentage was in 2008 (2.4%). The average per year was 1.57% for 12-year-old adolescents with at least one sealed tooth. Upon conducting the non-parametric trend test by year, it was observed that there was no statistically significant trend during this period (p=0.509). Table 1 and Figure 1 show the prevalence of caries (% DMFT>0) and the total sample in each year for the study period.

**Table 1:** Percentage distribution of 12-year-old adolescents with at least one tooth with PFS and DMFT experience The p-value was calculated using the nonparametric test for trends across ordered groups: p=0.509. *% of sealants=percentage of 12-year-old adolescents with at least one tooth with PFS. ^†^Caries prevalence in the age group of 10 to 14 years old. ^‡^Population of 12 years old. PFS, pit and fissure sealants; DMFT, decayed, missing, and filled teeth

Year	% of sealants*	%DMFT>0, 10-14^†^	N, 12 years old^‡^
2005	0.4	76.9	2714
2006	2.2	78.0	2992
2007	2.0	73.8	3269
2008	2.4	69.9	3547
2009	2.2	67.8	3758
2010	1.4	63.2	4245
2011	1.1	65.5	3769
2012	1.3	65.2	5391
2013	1.2	63.2	5072
2014	1.5	61.6	4882

**Figure 2:**
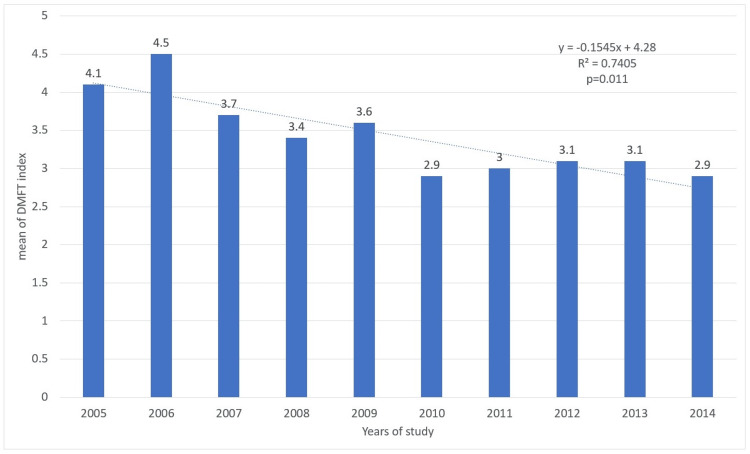
DMFT in adolescents aged 10 to 14 years by year in Mexico. The p-value was calculated using the nonparametric test for trend across ordered groups. Trend lines were generated in Excel, using the trend line format. DMFT, decayed, missing, and filled teeth

Figure 2 shows the average DMFT in Mexican adolescents aged 10 to 14 receiving health services by year. The average DMFT for the 2005 and 2014 period was 3.43. The lowest average DMFT was 2.9, in 2010 and 2014, while the highest was in 2006 (DMFT=4.5). Upon conducting the trend test by year, a statistically significant negative trend (p=0.011) was observed, indicating that as years went by, the average DMFT decreased (Figure 2).

**Figure 3:**
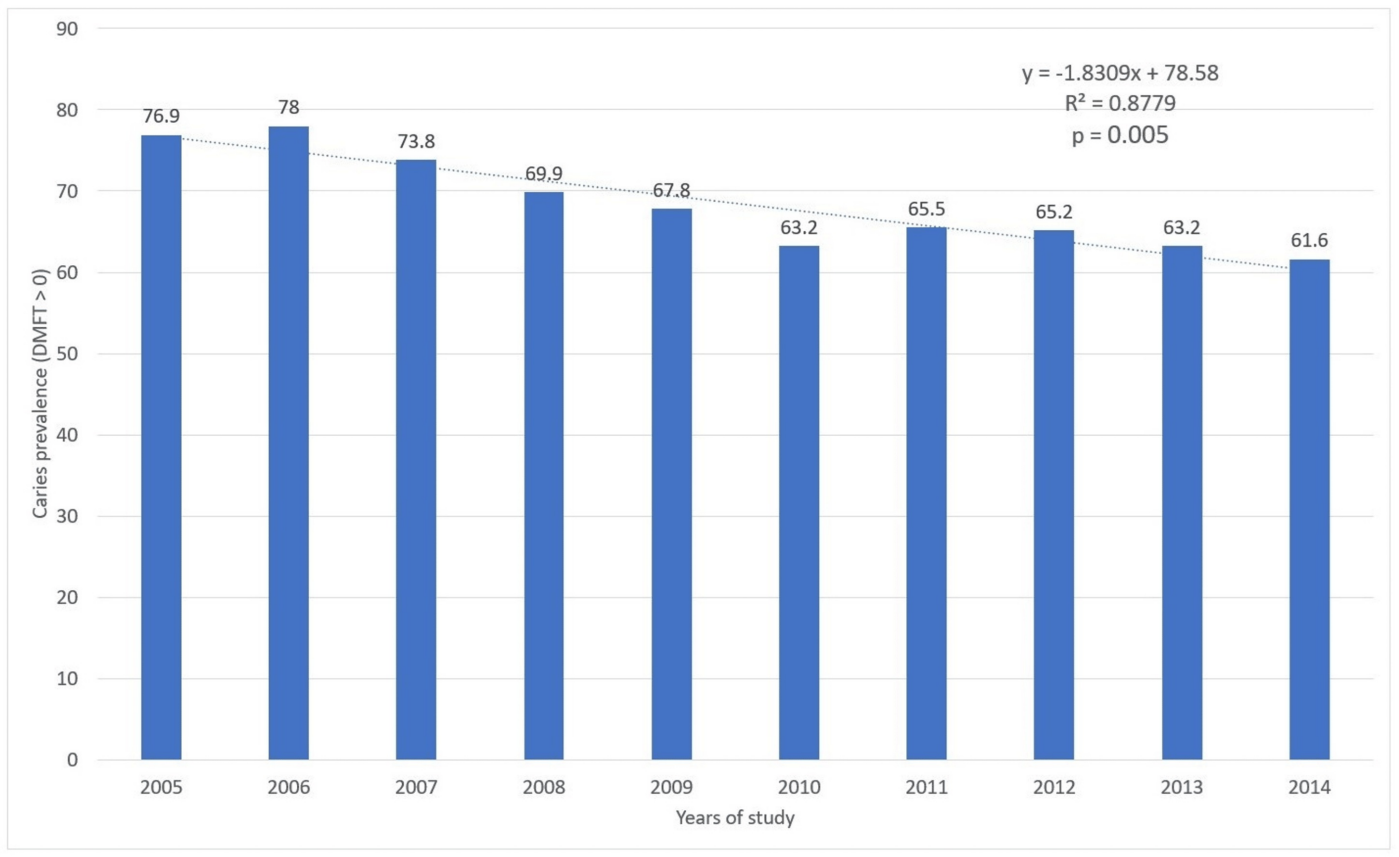
Caries prevalence (DMFT>0) in adolescents aged 10 to 14 years by year in Mexico. The p-value was calculated using the nonparametric test for trend across ordered groups. Trend lines were generated in Excel, using the trend line format. DMFT, decayed, missing, and filled teeth

Figure 3 shows the caries prevalence (DMFT>0) in Mexican adolescents aged 10 to 14 receiving health services by year. The caries prevalence between 2005 and 2014 was 68.5%, with the highest in 2006 (78.0%) and the lowest in 2014 (61.6%). The trend test by year showed a statistically significant negative trend (p=0.005) during this period, indicating that as years went by within this interval, the average of dental caries decreased (Figure 3).

**Figure 4:**
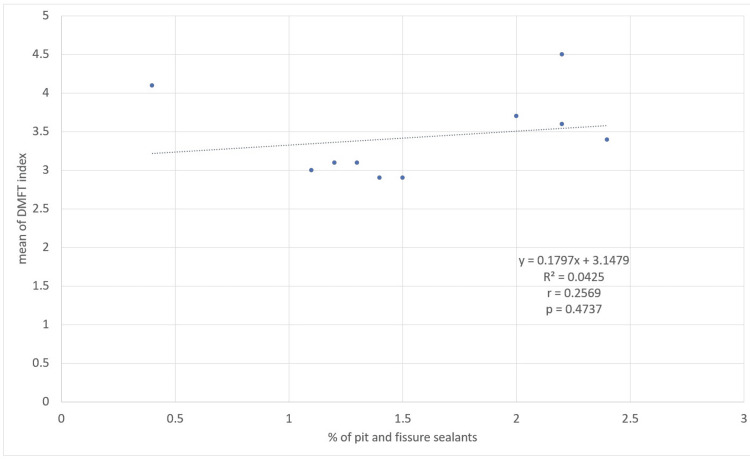
Correlation between the percentage of 12-year-old adolescents with at least one tooth with PFS and the average of DMFT in adolescents aged 10 to 14 years. The p-value was calculated using Spearman's rho test. Trend lines, equations, and R-squared values were generated in Excel, using the trend line format. PFS, pit and fissure sealants; DMFT, decayed, missing, and filled teeth

**Figure 5:**
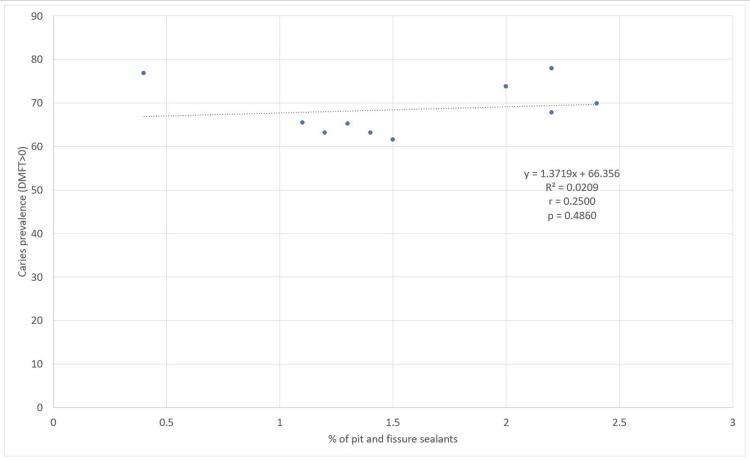
Correlation between the percentage of 12-year-old adolescents with at least one tooth with PFS and caries prevalence in adolescents aged 10 to 14 years. The p-value was calculated using Spearman's rho test. Trend lines, equations, and R-squared values were generated in Excel, using the trend line format. PFS, pit and fissure sealants; DMFT, decayed, missing, and filled teeth

No statistically significant correlation was observed in Spearman's correlation analysis between the percentage of 12-year-old adolescents with at least one tooth with PFS and the average DMFT (r=0.2569, p=0.4737) (Figure 4) or caries prevalence (r=0.2500, p=0.4860) (Figure 5) in children aged 10 to 14 years.

## Discussion

The present study aimed to ascertain PFS prevalence in Mexican adolescents who use publicly funded dental health services, and its correlation with caries indices between 2005 and 2014. The use of PFS was approximately 2.5%, and we did not find a relationship between the use of PFS and caries indices. The PFS use was low, compared to Puerto Rico (4.3%), Santo Tomé (2.2%), and Greece (8.0%8.3%) [26,29,30]. The prevalence of PFS is generally higher in more developed countries, such as the USA (31.3%), Italy (2.7%), China (3.7%), and Portugal (58.8%), where at least one PFS was applied [23,25,27,31]. Such variation may be associated with each country's socioeconomic context, caries levels among the population under scrutiny, availability of dental services, or even methodological differences in each study.

A concerning aspect of our results was that, in addition to the low PFS percentage generally, there was no trend toward an increase in PFS use. For comparison, a study conducted on Asian-American children in the USA from 2016 to 2020 found a decreasing trend in PFS use [32].

There was no correlation between PFS percentage and caries experience in our ecological study. The results of other studies using individual-level data are consistently different, showing a caries preventive effect of PFS [20-22]. Such disparate findings may be due to multiple reasons discussed here in terms of their importance. For example, a) ecological studies focus on populations rather than individuals and may not capture individual variability. Also, b) they might not have been able to control for confounding factors that could change the results, like self-care adherence, diet cariogenic potential, how often they go to the dentist, and other factors that could moderate the link between PFS and the number of carious lesions as well as their severity. Also, c) if there are multiple interventions or simultaneous changes in dental health policies, such transitions may be obscuring trends specific to PFS. Finally, d) differences in socioeconomic and geographic factors that exist throughout Mexico may influence caries prevalence and PFS use. With Mexico having a large and loosely organized dental profession, data from private care services are not centrally collected; we are exclusively looking at publicly funded PFS use.

The use of PFS as a preventive strategy in Latin America varies widely among countries, with only a few having comprehensive programs or data on the subject. For example: Costa Rica and Mexico have documented programs that specifically employ sealants for caries control in children, targeting populations at high risk​. In Brazil, sealants are primarily used in public health interventions for children in lower socioeconomic strata or rural areas, often as part of community-based initiatives. Chile has implemented school-based sealant programs that have been shown to be cost-effective in reducing caries among adolescents​. Limited studies from Colombia, Peru, and Venezuela report the sporadic use of sealants, typically as part of localized efforts or research-driven interventions rather than sustained national policies​. Overall, the use of sealants in Latin America is less prevalent compared to other regions such as North America and Europe. The implementation of these programs is generally influenced by economic disparities, the availability of resources, and the existence of organized national health policies [33].

On the other hand, the use of PFS for caries prevention in children and adolescents shows variability across regions, reflecting different implementation levels of preventive dental care. Globally, the trends indicate that sealants are highly effective in controlling dental caries, especially in permanent molars, and their use has been supported by evidence-based clinical guidelines from organizations like the American Dental Association (ADA). Sealants are recommended as a standard preventive intervention in countries like the United States, Canada, and various European nations, where national oral health programs have been established to promote their application in school-based settings. In many high-income countries, sealants are integrated into public health policies for children and adolescents, often targeting high-risk populations. For example, in the United States, sealant programs are a critical component of school-based oral health services, resulting in a steady increase in sealant use over the past few decades. Similarly, European countries have implemented national and regional initiatives to promote sealant use as part of comprehensive caries management strategies. In contrast, other countries show slower adoption, largely due to financial constraints, lack of resources, and limited access to dental care. In China, school-based sealant programs have been implemented with positive outcomes, but overall usage rates remain lower compared to Western countries. Sealant use in children is less common in lower-income nations due to the high cost of materials and limited access to dental professionals trained in these procedures [34]. Overall, global trends suggest that while the efficacy of sealants is well-established, disparities exist in their usage across different regions, with high-income countries showing broader and more consistent implementation compared to low- and middle-income nations [35].

The present study has limitations that need to be considered for proper interpretation of results. Methodologically, ecological studies present the risk of ecological fallacy, whereby DMFT and PFS data cannot be traced back to the individual. Moreover, the intensity of PFS delivery could modify the patterns we observed, as we consider a child to be PFS-"positive" if they have at least one PFS surface. More comprehensive sealing of occlusal surfaces in eligible posterior teeth may hold a different relationship with DMFT data. In ecological studies, the primary focus is on analyzing population-level data rather than individual-level data. Consequently, the lack of specific sample details, such as individual demographic information or health status, is a common and accepted limitation inherent to this study design. Ecological studies aim to explore trends and associations at a community, regional, or national level. As such, they are not designed to examine individual risk factors or detailed health outcomes. This aggregated approach allows researchers to detect broader patterns and generate hypotheses about the relationship between population-level interventions (e.g., public health policies or environmental exposures) and outcomes (such as changes in caries prevalence).

## Conclusions

The present ecological study in Mexico found that the PFS prevalence in the adolescent population was low. While there was no obvious trend suggesting an increase or decrease in PFS use over time, there was a decrease in the DMFT index during the same period. Given the strength of the census data obtained for the present retrospective analysis, such DMFT decline is unlikely to be primarily related to PFS use at the current level of intensity in publicly funded dental services. Our study will not only fill a gap in the existing literature by analyzing these trends in Mexico but will also provide a basis for improving public health policies aimed at reducing the burden of caries among adolescents, hopefully guiding preventive oral health strategies focused on accessible and equitable interventions.
